# Examining Predictors of Real-World User Engagement with Self-Guided eHealth Interventions: Analysis of Mobile Apps and Websites Using a Novel Dataset

**DOI:** 10.2196/11491

**Published:** 2018-12-14

**Authors:** Amit Baumel, John M Kane

**Affiliations:** 1 Department of Community Mental Health University of Haifa Haifa Israel; 2 Zucker Hillside Hospital Glen Oaks, NY United States

**Keywords:** eHealth, mHealth, user engagement, user experience, therapeutic alliance, persuasive design, behavior change

## Abstract

**Background:**

The literature suggests that the product design of self-guided electronic health (eHealth) interventions impacts user engagement. Traditional trial settings, however, do not enable the examination of these relationships in real-world use.

**Objective:**

This study aimed to examine whether the qualities of product design, research evidence, and publicly available data predict real-world user engagement with mobile and Web-based self-guided eHealth interventions.

**Methods:**

This analysis included self-guided mobile and Web-based eHealth interventions available to the public—with their qualities assessed using the Enlight suite of scales. Scales included Usability, Visual Design, User Engagement, Content, Therapeutic Persuasiveness, Therapeutic Alliance, Credibility, and Research Evidence. Behavioral data on real-world usage were obtained from a panel that provides aggregated nonpersonal information on user engagement with websites and mobile apps, based on a time window of 18 months that was set between November 1, 2016 and April 30, 2018. Real-world user engagement variables included average usage time (for both mobile apps and websites) and mobile app user retention 30 days after download.

**Results:**

The analysis included 52 mobile apps (downloads median 38,600; interquartile range [IQR] 116,000) and 32 websites (monthly unique visitors median 5689; IQR 30,038). Results point to moderate correlations between Therapeutic Persuasiveness, Therapeutic Alliance, and the 3 user engagement variables (.31≤*r*s≤.51; *P*s≤.03). Visual Design, User Engagement, and Content demonstrated similar degrees of correlation with mobile app engagement variables (.25≤*r*s≤.49; *P*s≤.04) but not with average usage time of Web-based interventions. Positive correlations were also found between the number of reviews on Google Play and average app usage time (*r*=.58; *P*<.001) and user retention after 30 days (*r*=.23; *P*=.049). Although several product quality ratings were positively correlated with research evidence, the latter was not significantly correlated with real-world user engagement. Hierarchical stepwise regression analysis revealed that either Therapeutic Persuasiveness or Therapeutic Alliance explained 15% to 26% of user engagement variance. Data on Google Play (number of reviews) explained 15% of the variance of mobile app usage time above Enlight ratings; however, publicly available data did not significantly contribute to explaining the variance of the other 2 user-engagement variables.

**Conclusions:**

Results indicate that the qualities of product design predict real-world user engagement with eHealth interventions. The use of real-world behavioral datasets is a novel way to learn about user behaviors, creating new avenues for eHealth intervention research.

## Introduction

### Background

Self-guided electronic health (eHealth) interventions have the potential to increase access to evidence-based care, while reducing the costs associated with service uptake [1,2]. The impact of these interventions, however, is limited by the ability to engage users in therapeutic activities and to support users’ adherence to the therapeutic process [3,4]. As eHealth interventions require individuals to engage with self-care outside of traditional health care settings [5-7], individuals’ engagement must compete with other events in their daily lives and fluctuating motivation to engage in effortful behavior [8]. As a result, user engagement with mobile apps and websites across the behavior change spectrum is low in the absence of human support [9].

There is a body of literature examining how intervention design may facilitate engagement and behavior change [10-14]. For example, systematic reviews have found relationships between the incorporation of therapeutic persuasiveness (ie, persuasive design and behavior change techniques) into the eHealth intervention and user adherence [15] and the intervention’s efficacy [16]. Studies have also shown that the incorporation of conversational agents within self-guided eHealth interventions impacts user engagement [17,18], suggesting that conversational agents enhance the relational factors within the program—factors that are part of the therapeutic alliance fostered between the user and the program [19-21]. Although these studies provide convincing evidence linking product design to user engagement, the understanding of these relationships is still limited by our ability to examine user engagement in the real world, while comparing large numbers of products within the same study.

### Comparing a Large Number of Products

Certain methodologies enable the cost-effective comparison of a large number of interventions. The Multiphase Optimization Strategy, for example, offers a paradigm to incorporate a randomized experimentation in a way that directly compares many different intervention components to identify the group of active components leading to the best outcome [22]. When it comes to product designs that are largely different in their functionalities, however, to control all the active components, the different functions would have to be available within the utilized digital platform—a process that would be highly expensive given the average development price of a single health app [23].

A novel way to compare large numbers of digital products is to utilize datasets that record user behaviors on a large number of websites and mobile apps. This approach can enable the documentation of variance in user engagement across a wide range of product designs using the same analytical framework. The big data commonly generated and stored by digital platforms can be used to learn about user behaviors in order to refine conceptual models or theories, and to understand processes related to eHealth interventions [24]. Additionally, using commonly generated data enables us to record real-world user behaviors outside of study settings that involve interactions with research staff—interactions that might impact user engagement [25,26].

### Identifying the Different Quality Aspects of Product Design

To utilize commonly generated data and to understand their relationship with eHealth product design, there is also a need to identify the different aspects of product design in a reliable way. The literature suggests several approaches to evaluating eHealth product design (see the study by BinDhim et al [27] for a review). These include using a predefined list of what the app should contain, assessing the inclusion of evidence-based content, assessing the usability of predefined app functions, using consumer reviews and ratings, and utilizing criteria-based rating scales. Criteria-based rating scales rest on a heuristic evaluation approach—a method that has been broadly researched and used for assessing eHealth and technology products [28-30]. Heuristics are broad principles of product design that can be inspected by evaluators before empirical testing. The advantage of heuristic evaluation is that it enables the cost-efficient identification of design problems without the need for a predefined list of features that may exclude the users’ experience of utilizing the product [29,31,32]. Criteria-based rating scales are based on core concepts, each comprising different heuristics, which are used by trained raters to objectively examine and score the quality of eHealth programs [12,33-35]. In the absence of such clearly defined rating systems, scoring tends to be less reliable [36].

Enlight is a suite of criteria-based rating scales that was used in this study. It covers 11 different quality constructs and checklists (eg, Usability and Credibility) that were produced by trained raters. Enlight is the first suite of measures to incorporate behavior change, persuasive design, and therapeutic alliance concepts—concepts that have been found to affect a program’s therapeutic potential [15,16]. As the tool shows high inter-rater reliability scores at the construct level, it enables us to use it to examine the relationships between different aspects of product design and metrics of user engagement.

Within this context, Baumel and Yom-Tov conducted a preliminary investigation, examining the correlations between 6 quality aspects of product design and real-world user engagement with 30 Web-based programs [37]. Real-world user engagement was based on a proprietary dataset of anonymized logs from consenting users of a Microsoft Internet Explorer add-on. The quality scores were generated using Enlight [21]. In the preliminary study, it was found that product quality ratings predicted which Web-based interventions were more engaging and in particular that Therapeutic Persuasiveness was the most robust predictor of user adherence (ie, duration of use, number of unique sessions; 40≤*r*s≤.58; *P*s≤.005) [37].

Although these findings were novel in terms of the methods applied, they were limited in several aspects. First, the dataset only included Web-based interventions, whereas the literature suggests that there has been a massive increase in mobile phone ownership and mobile health app usage in recent years [38]. Second, as the analysis was based on a small sample of interventions, the question arises as to whether the same pattern of results will be found with a larger dataset, enabling identification of correlations with other aspects of product design. Third, the study did not incorporate important metrics that relate to research evidence, a product’s credibility, and publicly available data on user acceptance (eg, user ratings on app stores). Examining the relationship between research evidence and real-world uptake is key in light of the notion that efficacy trials largely emphasize internal validity over real-world issues, such as the technological environment, implementation, and sustainability, and thus may not provide the needed validation [26]. Furthermore, to the best of our knowledge, there has been no study linking the credibility of the source that developed the program or user ratings on app stores *and* real-world user engagement with eHealth programs.

### This Study

The aims of this study were, therefore, to fill this gap in the literature by (1) examining whether different qualities of product design predict real-world user engagement with both mobile- and Web-based self-guided eHealth interventions; (2) exploring the associations between scale items, data, and real-world user engagement; (3) examining whether research evidence and product credibility metrics are associated with real-world user engagement; (4) examining the associations between publicly available data on program acceptance (eg, star ratings on app stores) and real-world usage and whether these data enhance the prediction of user engagement above expert ratings; and (5) establishing Enlight’s validity in predicting user engagement with eHealth interventions based on a large and independent dataset of user behaviors.

## Methods

### Selection of Interventions

We screened for eligibility all eHealth programs that were assessed based on Enlight suite of scales between September 2016 and December 2017—during the scale development phase and afterward as part of a nonprofit project aimed at evaluating the quality of eHealth programs [39]. The clinical aims of the selected programs were broad, spanning the behavioral health domain. These programs could be grouped into those targeting mental health (eg, depression, anxiety, and well-being) and those targeting health-related behaviors (eg, diet, physical activity, and smoking and alcohol cessation).

The sources of the eHealth programs that were screened for eligibility are presented in Table 1. A total of 84 programs were randomly selected and rated between September and December 2016, following a systematic identification process, conducted as part of Enlight tool’s development [21]. In this process, we created a list of mobile apps and Web-based programs through keyword searches in Google Play and Google search engine (this systematic process and a complete list of keywords is presented in Multimedia Appendix 1). Following the tool’s development phase, we used a similar systematic identification process—that now also included paid programs—to rate additional programs between January and December 2017. All programs found in the top 10 Web or mobile app search results (and that had not been rated before) were rated, reaching a number of additional 50 eHealth programs. Finally, 8 eHealth programs were identified based on recommendations from eHealth researchers and product developers (eg, the Digital Behavioral Health Workgroup at Northwell Health). This selection process yielded a list of 142 programs, among which, 21 programs had both mobile and Web-based versions. As the behavioral data on program usage and our inclusion criteria in this study relate separately to websites and mobile apps, we eventually screened for eligibility 71 Web-based programs and 92 mobile apps.

### Inclusion and Exclusion Criteria

To be included in this analysis, interventions had to be (1) self-guided and (2) cost-free. Apps that were free to install, yet had a trial period (ie, paid with free trial) were also included; however, we examined their impact on results using a sensitivity analysis, as will be further described in the data analysis section. Exclusion criteria included (1) programs that were only trackers, as their quality ratings do not rely on the full list of product ratings covered in Enlight; (2) websites that were not focused mostly on the intervention itself (eg, websites with many blog articles that did not require user log-in)—as the data on the platform are provided in an aggregated way that does not distinguish between the website’s different Web pages; (3) apps that did not have an Android version, as the behavioral dataset did not include information on iOS apps; and (4) programs without usage data in our behavioral dataset (due to, for example, small number of users).

### Measures

#### Enlight Quality Ratings

Enlight is a comprehensive suite of criteria-based measurements—completed by trained raters who review the eHealth program—that was developed by a multidisciplinary team through a rigorous process of content analysis, grouping, and classification of 476 identified criteria items [21,40]. The tool covers 6 different product quality domains: Usability, Visual Design, User Engagement, Content, Therapeutic Persuasiveness (ie, persuasive design and incorporation of behavior change techniques), and Therapeutic Alliance (see Multimedia Appendix 2 for a detailed description of the scales used and operational definitions of all items). Each quality domain score ranges from 1 to 5 and is based on averaging the criteria ratings produced by the raters on a Likert scale (ranging from 1 to 5). For example, Therapeutic Persuasiveness is calculated by averaging the raters’ scores of the following criteria items: call to action (eg, goal settings, prompting goals, and encouragement to complete goals), load reduction of activities (eg, set graded tasks), therapeutic rationale and pathway (eg, reasoned action and provide information about behavioral health link), rewards (eg, contingent rewards), real data-driven/adaptive content (eg, self-monitoring of behavior), ongoing feedback, and expectations and relevance.

**Table 1:** Sources of previously rated eHealth programs that were screened for eligibility in this study.

Source	eHealth programs (N=142), n (%)	Web-based program (N=71), n (%)	Mobile app (N=92), n (%)	Programs with 2 delivery mediums^a^ (N=21), n (%)
Systematic review: programs randomly selected and rated during Enlight’s development phase	84 (59.2)	43^b^ (61)	49^b^ (53)	8 (38)
Additional programs found in the top 10 mobile or Web search results of the systematic identification process	50 (35.2)	25 (35)	38 (41)	13 (62)
Additional programs that were identified based on personal recommendations	8 (5.6)	3 (4)	5 (5.4)	0 (0)

^a^The 2 delivery mediums are (1) Web-based programs and (2) mobile apps.

^b^In the original study, we examined 42 mobile apps and 42 Web-based programs. Eventually, 7 websites and 1 mobile app had a similar version in the other delivery medium (mobile or website).

Enlight covers 2 additional measures that were included in this analysis. Credibility consists of a checklist calculated by aggregating the scores received in each of its respective categorical items (owners’ credibility, maintenance, strong advisory support, third party endorsement, and evidence of successful implementation). The checklist differs from the product quality assessments in 2 ways: (1) most credibility items cannot be rated before product deployment (as they rely on data that are collected afterward) and (2) the criteria included in the checklist are not expected to directly impact the end users’ experience of the product’s efficacy (however, they may expose the user to acknowledged risks or benefits). We also included an evidence-based program scale (ie, research evidence) that assesses the quality of the empirical research supporting the program within the current zeitgeist on a scale from 1 (very poor) to 5 (very good) [21].

A total of 3 individuals with clinical experience (eg, clinical psychologists) and with at least 1 year experience working in the eHealth domain (eg, content writing of eHealth programs and health technology coaching) performed the ratings, with 2 of them independently rating each program. Their training included a review of Enlight items, individual ratings of 7 eHealth programs that included group-solicited feedback on ratings, and then testing the ratings based on 5 additional programs (for a detailed review, see the study by Baumel et al [21]). In this study (post-training), the Enlight quality scales exhibited excellent inter-rater reliability scores between the 2 raters (intraclass correlations=.74 to .98, median .86).

#### Behavioral Data on User Engagement in the Real World

Information on user behaviors was obtained from SimilarWeb’s Pro panel data [41]. The panel provides aggregated nonpersonal information on user engagement with websites and mobile apps all over the world to enable Web and mobile app traffic research and analytics. It is based on several sources of anonymized usage data, such as that obtained from consenting users of mobile apps or browser add-ons (ie, products). A dedicated product team at SimilarWeb is responsible for building and partnering with hundreds of high-value consumer products that make up the panel. According to SimilarWeb, the products are used across diverse audiences, without cluttering the user with advertisements. Although benefiting from the products, users contribute to the panel, as they enable to document their online or mobile apps’ usage activities seamlessly and anonymously [41]. The data are not used by SimilarWeb or provided to any third parties for the purposes of marketing, advertising, or targeting any individual subjects. The data-gathering procedures comply with data privacy laws, including the way the data are collected, anonymized, stored, secured, and used. These procedures are updated regularly based on evolving data privacy legislation and requirements, such as the European Union’s General Data Protection Regulation [42].

The study was approved by University of Haifa Institutional Review Board. The measures were set to include data gathered over an 18-month period from November 1, 2016 to April 30, 2018. To examine user engagement with mobile apps, we used 2 measures retrieved from SimilarWeb Pro: (1) average app usage time (of all users) and (2) the percentage of users who downloaded the app and were still using it 30 days later. As this information is provided separately per country, we examined the country with the most app downloads. User engagement with the Web-based interventions was calculated based on average monthly visits to the website multiplied by the average visit duration.

To test the reliability of the data for the remaining websites, we calculated the Spearman correlation between Web usage time obtained from the SimilarWeb platform and the usage time calculated for websites in our previous work with Microsoft Research [37]. A strong positive correlation (*r*=.69; *P*<.001) was found between the 2 datasets for the 17 websites that had data on both platforms. In light of the difference between the 2 datasets (our previous work was focused only on Explorer browser users), we also examined the Spearman correlation between website global popularity ranks (ie, a rank that reflects the extent to which a website is utilized all over the world) on SimilarWeb platform and Alexa [43]—an independent source of information on user traffic. A very strong Spearman correlation was found (n=28; *r*=.78; *P*<.001). Relating to the validity of mobile app usage data, an Oath researcher [44] (RW) examined 30 randomly selected mobile apps with data on SimilarWeb and Oath’s own independent records of mobile app usage data. The researcher examined the correlation between the average number of user sessions per day in the 2 datasets, finding a very strong Spearman correlation (n=30; *r*=.77; *P*<.001). These findings suggested that there was sufficient convergent validity, which can be claimed if the correlation coefficient is above .50, although a value above .70 is usually recommended [45].

Finally, we documented publicly available data that relate to a program’s acceptability. The reported number of installs, average star ratings, and number of reviews were obtained from the Google Play store. Websites’ total monthly visits were obtained from the publicly available version of SimilarWeb.

#### Data Analysis

Medians and interquartile ranges (IQRs) were used to present the distribution of the variables. Pearson correlations were used to examine the relationships between the continuous variables. As Pearson correlations and linear regressions assume a normal distribution of the variables, we examined the skewness and kurtosis of the variables within acceptable limits of ±2 and performed a logarithmic transformation (base 10) of these variables [46,47]. This transformation was eventually applied to the following variables: Credibility, Research Evidence, number of reviews on Google Play, mobile app usage time, and website’s total monthly visits.

A sensitivity analysis was conducted to examine whether the pattern of correlations found differed depending on (1) the clinical aim of the intervention (ie, mental health or health-related behaviors) and (2) whether paid programs with a free trial were included or excluded. The differences between correlations of these groups were calculated using Fisher Z-transformation. Independent sample *t* tests with Bonferroni correction were performed to examine the difference between categorical items on the behavioral measures.

Finally, a series of hierarchical stepwise linear regressions was applied to examine the ability to predict user engagement in the real world independent of empirical testing. In the first step, we examined the percentage of variance explained by Enlight quality ratings. In the second step, we examined whether empirical data on real-world usage that were available at no cost (eg, Google Play for mobile apps: number of downloads, star reviews, and number of reviews; SimilarWeb for websites: monthly visits) significantly increased the explained variance. In each of these steps, a stepwise approach was applied to avoid adding predicting variables that did not contribute significantly to the overall model.

## Results

**Figure 1:**
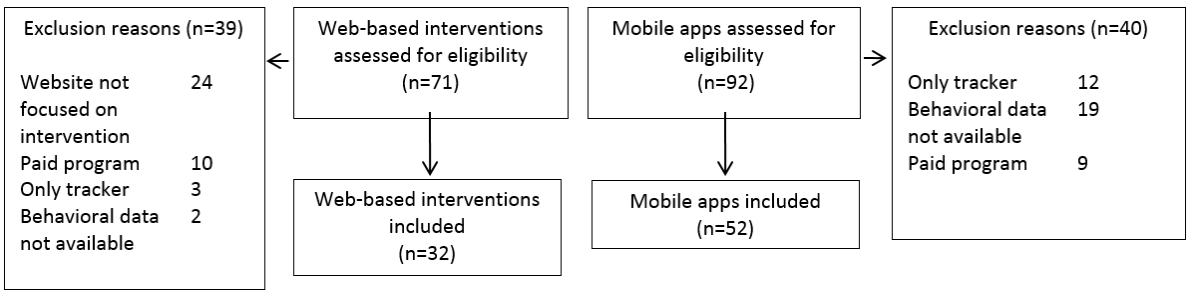
Flow diagram of program selection.

The analysis included user behavior data involving 52 mobile apps and 32 websites (see Figure 1 for the flow diagram of program selection and Multimedia Appendix 3 for a full list of included programs). Overall, 9 programs had usage data for both Web- and mobile-app versions, reaching a total of 75 different programs with data. Within the 18-month time window of the analysis, the 52 mobile apps had a median of 38,600 downloads (IQR 116,000), and the 32 Web-based interventions had a median of 5689 monthly unique visitors (IQR 30,038). The median monthly usage time was 5.11 min (IQR 20.51) for mobile apps and 9.0 min (IQR 7.37) for websites. The medians and IQRs of Enlight product quality ratings were as follows: Usability median 3.66 (IQR 1.66); Visual Design median 3.00 (IQR 1.00); User Engagement median 3.20 (IQR 1.10); Content median 3.50 (IQR 1.00); Therapeutic Persuasiveness median 2.71 (IQR 1.00); and Therapeutic Alliance median 2.66 (IQR 1.33).

Pearson correlations between Enlight product quality ratings, Credibility, and Research Evidence are presented in Table 2. User Engagement, Content, Therapeutic Persuasiveness, and Therapeutic Alliance were positively correlated with Credibility (.26≤*r*s≤.51, *P*s≤.01) and Research Evidence (.21≤*r*s≤.39, *P*s≤.04). That is, the interventions with higher scores in these quality domains had higher Credibility and Research Evidence ratings.

Pearson correlations between Enlight quality ratings and items, Credibility and Research Evidence, publicly available data on program acceptance, and the 3 behavioral variables are presented in Table 3. Results point to moderate positive correlations between Therapeutic Persuasiveness, Therapeutic Alliance, and the 3 behavioral variables (.31≤*r*s≤.51, *P*s≤.03). Visual Design, User Engagement, and Content had a similar degree of correlations with the user engagement variables of mobile apps (.25≤ *r*s≤.49, *P*s≤.04), but not Web-based interventions.

Altogether, a similar pattern of results was found between the criteria items of domains with significant correlations and the respective behavioral variables. It is worth noting that 3 criteria items of the Therapeutic Persuasiveness domain—rewards, data-driven/adaptive, and ongoing feedback—showed significant correlations with mobile app user retention after 30 days; however, these items did not correlate significantly with mobile app usage time. In terms of the Enlight checklists, a program’s Credibility had positive correlations with the usage time of apps (*r*=.34; *P*=.006) and websites (*r*=.30; *P*=.04), but no significant correlations were found between Research Evidence and the 3 behavioral variables. Finally, results point to positive correlations between the number of reviews on Google Play and average app usage time (*r*=.58; *P*<.001) and user retention after 30 days (*r*=.23; *P*=.049). The number of installs and average star reviews had significant positive correlations with mobile app usage time (*r*s=.25 and .36, respectively; *P*s=.04 and .005, respectively).

As the Credibility checklist covers different independent categorized items, a series of independent sample *t* tests with Bonferroni correction was performed to examine the difference between Credibility items (Owners’ Credibility, Maintenance, Strong Advisory Support, Evidence of Successful Implementation) in terms of the usage time of mobile and Web-based interventions. (Third Party Endorsement was not included in this test as a relevant party had endorsed only a few programs.) A significant difference was found in the average usage time of mobile apps, favoring programs that had Evidence of Successful Implementation (ie, a high number of downloads or positive reviews) *t*_50_=3.88, *P*<.001, Cohen *d*=1.07, and in maintenance, favoring programs that had been updated within the previous 6 months, *t*_50_=2.63, *P*=.28, Cohen *d*=0.73. As both variables relied on the data documented on Google Play, we added them in the second step of the regression analysis described below.

**Table 2:** Pearson correlations between Enlight product quality ratings, Credibility, and Research Evidence metrics (n=75).

Scale	Credibility		Research evidence	
	*r* ^a^	*P* value	*r*	*P* value
Usability	.02	.45	.00	.50
Visual Design	.08	.24	−.13	.14
User Engagement	.39^b^	<.001	.21^b^	.03
Content	.51^b^	<.001	.39^b^	<.001
Therapeutic Persuasiveness	.26^b^	.01	.21^b^	.04
Therapeutic Alliance	.39^b^	<.001	.26^b^	.01

^a^Using Fisher Z-transformation, no significant differences in Pearson correlation values were found between programs targeting mental health (n=51) and those targeting health-related behaviors (n=24).

^b^Indicates significant correlations.

**Table 3:** Pearson correlations between Enlight scales, publicly available data on program acceptance, and real-world user engagement with eHealth interventions.

Scale		Mobile app interventions (n=52)				Web interventions (n=32)	
		Usage time		User 30-day retention		Usage time	
		*r* ^a^	*P* value	*r*	*P* value	*r*	*P* value
**Usability**		.15	.14	−.07	.32	.01	.48
	Ease of use	.21	.07	.05	.36	.10	.29
	Learnability	.07	.31	−.13	.18	−.14	.22
	Navigation	.13	.18	−.11	.22	.03	.43
**Visual Design**		.31^b^	.01	.25^b^	.04	.09	.32
	Aesthetics	.28^b^	.02	.28^b^	.02	.12	.26
	Layout	.32^b^	.01	.19	.09	.14	.22
	Size	.24^b^	.047	.18	.10	−.00	.49
**User Engagement**		.49^b^	<.001	.39^b^	.002	.21	.12
	Captivating	.51^b^	<.001	.31^b^	.01	.38^b^	.02
	Content presentation	.53^b^	<.001	.29^b^	.02	.22	.12
	Interactive	.13	.19	.25^b^	.04	.28	.06
	Not irritating	.23	.07	.17	.15	−.11	.31
	Targeted/tailored/personalized	.41^b^	.001	.38^b^	.003	.01	.48
**Content**		.45^b^	<.001	.33^b^	.009	.16	.19
	Evidence-based content	.43^b^	.001	.31^b^	.01	.15	.20
	Information provision quality	.35^b^	.006	.34^b^	.006	−.01	.48
	Complete and concise	.39^b^	.002	.35^b^	.005	.27	.07
	Clarity about program’s purpose	.43^b^	.001	.10	.23	.09	.30
**Therapeutic Persuasiveness**		.36^b^	.004	.39^b^	.002	.33^b^	.03
	Call to action	.34^b^	.007	.49^b^	<.001	.29	.055
	Load reduction of activities	.44^b^	.001	.37^b^	.004	.37^b^	.02
	Therapeutic rationale/pathway	.47^b^	<.001	.36^b^	.004	.21	.12
	Rewards	.14	.16	.26^b^	.03	.22	.12
	Data-driven/adaptive	.11	.21	.24^b^	.046	.23	.10
	Ongoing feedback	.14	.16	.33^b^	.01	.14	.23
	Expectations and relevance	.31^b^	.01	.03	.41	.19	.15
**Therapeutic Alliance**		.51^b^	<.001	.31^b^	.01	.36^b^	.02
	Basic acceptance and support	.45^b^	<.001	.40^b^	.002	.42^b^	.009
	Positive therapeutic expectations	.45^b^	<.001	.33^b^	.009	.22	.12
	Relatability	.40^b^	.002	.09	.26	.32^b^	.04
**Enlight postempirical measures**							
	Credibility	.34^b^	.006	−.03	.42	.31^b^	.04
	Research evidence	.08	.28	−.13	.18	.19	.15
**Google Store data**							
	Number of installs	.25^b^	.04	.10	.25	N/A^c^	N/A
	Average star reviews	.36^b^	.005	.18	.11	N/A	N/A
	Number of reviews	.58^b^	<.001	.23^b^	.049	N/A	N/A
**Web traffic data**							
	Total monthly visits	N/A	N/A	N/A	N/A	.19	.15

^a^Using Fisher Z-transformation, no significant differences in Pearson correlation values were found between programs targeting mental health (mobile apps n=38 and websites n=22) and those targeting health-related behaviors (mobile apps n=14 and websites n=10) or between the full samples and the samples after subtracting paid programs with free trials (n=7).

^b^Indicates significant correlations.

^c^N/A: not applicable.

**Table 4:** Hierarchical stepwise regressions for predictors of user engagement with self-guided mobile and Web-based interventions.

Variable			B^a^	SE B	Beta	*P* value	*R*^2^ change	*P* value
**Mobile app usage time (n=52)**								
	**Step 1**							
		Therapeutic Alliance	.47	.11	.51	<.001	.26	<.001
	**Step 2**							
		Therapeutic Alliance	.29	.11	.31	.02	.15	.001
		Number of reviews	.29	.08	.44	.001	N/A^b^	N/A
**Mobile app 30-day retention (n=52)**								
	**Step 1**							
		Therapeutic Persuasiveness	.01	.01	.39	.004	.15	.004
	Step 2^c^		N/A	N/A	N/A	N/A	N/A	N/A
**Web intervention total usage time (n=32)**								
	**Step 1**							
		Therapeutic Alliance	2.87	1.34	.36	.04	.13	.04
	Step 2^c^		N/A	N/A	N/A	N/A	N/A	N/A

^a^B: unstandardized regression coefficient.

^b^N/A: not applicable.

^c^No new variables were entered into the equation.

Table 4 presents the hierarchical stepwise regressions performed to examine the predictors and percentages of real-world user engagement variance that were explained by Enlight product quality ratings and whether publicly available data on program acceptance added to the explained variance. The analysis showed that the variance of user engagement explained by Enlight ratings ranged between 13% and 26%. In each of the first steps, following the entrance of one Enlight quality rating—either Therapeutic Persuasiveness or Therapeutic Alliance—no other metric was found to significantly contribute to the model. The analysis also showed that publicly available data (number of reviews) explained 15% of the variance of mobile app usage time above Enlight ratings; publicly available data did not significantly explain the variance of mobile app user retention or Web-based intervention usage time.

## Discussion

### Principal Findings

This study presents novel findings about the relationship between product design and user engagement with self-guided eHealth interventions in the real world. We found that product quality in terms of Visual Design, User Engagement, Content, Therapeutic Persuasiveness, and Therapeutic Alliance was positively correlated with real-world usage of mobile apps. Therapeutic Persuasiveness and Therapeutic Alliance were also positively correlated with real-world usage of Web-based programs. Similar to previous findings, Visual Design, User Engagement, and Content were not found to have a significant correlation with usage time of Web-based interventions [37]. Although the domains’ criteria items had similar patterns of correlation with the behavioral variables, 3 Therapeutic Persuasiveness items—rewards, data-driven/adaptive, and ongoing feedback —showed significant correlations with mobile app user retention after 30 days but no significant correlations with mobile app usage time. It might be that these variables, which examine the ongoing reciprocal interaction between the software and the user, are sensitive to the time window of use rather than to the accumulated usage time. However, future research should first be conducted to examine whether this pattern of results is replicated.

Congruent with our previous examination [37], Usability was not associated with the behavioral variables of user engagement. It is important to note that as our analysis was based on between-program evaluation, it does not mean that improving program’s usability will not enhance user engagement with the program. Alternatively, we suggest that using the Usability score to compare *different* programs might not capture which programs are more engaging to users. Therefore, Usability should be considered to be a barrier rather than a facilitator of user engagement [48,49]. A future direction could be to define ranges—in terms of Usability scores—that identify the point at which a program is usable enough to be evaluated based on other metrics.

Our analysis of Google Play data revealed positive correlations between the number of reviews, mobile app usage time, and user retention; number of installs and average star reviews were also positively correlated with mobile app usage time. These findings present a link between data available to the public on app stores and an app’s overall tendency to engage users. Regression results, however, suggested that these data do not always enhance our understanding of user engagement after accounting for the quality of product design—which could be determined before real-world use of the program. Hence, it could be informative to examine how expert-based rating tools such as Enlight can be used by developers during a program’s development phase to guide the process of product design before empirical testing.

Our analysis also showed that programs with better design quality had better research evidence. At the same time, research evidence did not predict user engagement in real-world use. This finding is congruent with Fleming et al’s study that examined published reports on the real-world uptake of eHealth programs [50]. The researchers found that indications of completion or sustained use (ie, completion of all modules or the last assessment or continuing to use apps after 6 weeks or more) varied from 0.5% to 28.6%, concluding that actual usage may vary from that reported in trials. Accordingly, our analysis implies that when research evidence supports a certain program, it does not necessarily mean that users will engage with the program in the real world. It is important to note that there may be many reasons for what could be referred to as “trial versus real-world gap,” including the impact of the study setting on user engagement [25] and populations being targeted for the study that differ from those using the program in the real world [26]. Future research should to be conducted to empirically examine this phenomenon and the factors influencing it.

It is also important to note that the analysis did not reveal an association between the Credibility items—developer’s credibility (eg, academic institute) and strong advisory support group—and user engagement variables. One reason that could explain this finding is the high costs associated with app development [23], which may create barriers for teams that are more focused on answering academic research questions based on grant funding. However, this explanation should be further examined to draw firm conclusions.

Finally, from a methodological perspective, the notion that stands behind the development of criteria-based rating tools is that in the absence of proper training, proper expertise, and proper use of developed scales, inter-rater agreement will be low, and therefore, the examination will not be reliable. For example, in a study to examine inter-rater reliability of mobile health (mHealth) app rating measures, Powell et al gathered a panel of 6 reviewers to review the top 10 depression apps and top 10 smoking cessation apps from the Apple iTunes App Store and evaluate them based on several measures. The authors found a wide variation in the inter-rater reliability of measures, with some measures being more robust across categories of apps than others [36]. In recent years, several studies have demonstrated that it is feasible to achieve sufficient inter-rater reliability with criteria-based rating scales by using trained raters [35,51]. To perform the ratings for this study and previous evaluations using the Enlight tool, raters had to complete a certain level of training to provide reliable ratings. This notion has been acknowledged for decades within the psychological assessment field ([52,53]; George et al, unpublished data, 1985) we hope that as the eHealth evaluation field moves forward, more attention will be paid to the proper use of methods to train and examine evaluators’ work.

### Limitations

This study has several limitations. First, the findings are not based on an experimental procedure that compared different designs of the *same* intervention. However, it would not be possible to utilize an experimental procedure to compare many aspects of product design at the same time. Consequently, if an association is not found between a quality domain and program usage, it does not mean that improving the program in this domain will not influence usage. Instead, it means that when comparing *different* programs, some aspects of quality are more important than others in predicting usage time. Second, this study examined user engagement, which is not the same as efficacy. Data suggest that there is a strong relationship between engagement and efficacy [54-56]; however, more does not always equal better [57]. A future research direction would be to measure efficacy using a large sample of programs “in the real world” and to examine the correlations between product design and efficacy. This testing should take into account fundamental questions related to participant consent and how to measure intervention outcomes.

### Conclusions

Results indicate that Enlight is a valid tool for predicting real-world user engagement with eHealth interventions, based on expert evaluations that were conducted before empirical testing with end users. The link between expert reviews and user behaviors in the real world supports the importance of rating tools that may enable trained experts to (1) guide the design of evidence-based interventions before testing with end users and (2) provide important details about the product’s potential to enable end users to make educated decisions when searching for self-guided interventions. Such details are presented in MindTools.io [39], a nonprofit website that publishes in-depth app reviews using Enlight rating scales.

Finally, the use of real-world behavioral datasets, which are garnered from a massive number of users, is a novel way to learn about user behaviors, creating new avenues of research and advancing our understanding of eHealth interventions. More studies are needed to shed light on the relationships between real-world uptake and data that emerge from other sources of information.

## Appendix

Multimedia Appendix 1 Description of the systematic identification process.

Multimedia Appendix 2 Description of scales and scale items.

Multimedia Appendix 3 Included programs.

## Abbreviations

eHealth: electronic health
IQR: interquartile range
mHealth: mobile health
