# Supplementary material for: Examining Predictors of Real-World User Engagement with Self-Guided eHealth Interventions: Analysis of Mobile Apps and Websites Using a Novel Dataset
Source: J Med Internet Res. 2018 Dec 14;20(12):e11491. doi: 10.2196/11491 (PMC6315225; doi:10.2196/11491)
Supplement: Multimedia Appendix 1 [file jmir_v20i12e11491_app1.pdf]

Multimedia Appendix 1 - Description of the Systematic Identification Process

The systematic identification of relevant programs followed the PRISMA statement guidelines [1]. For health-related behaviors, we targeted behaviors considered to be among the leading preventable causes of death due to chronic medical conditions in the United States [2]: diet, physical activity, and smoking and alcohol cessations. For mental health, we focused our search on the terms depression, anxiety, mental health, and well-being. (A complete list of search keywords is presented below the systematic identification process description.)

To identify relevant mobile applications, we conducted a systematic search of the Google Play store on September 5, 2016, by using search terms relevant for each condition (e.g., diet: diet or weight loss). The inclusion criteria for mobile applications were: (1) English language; (2) free of charge; and (3) from Android categories "Health & Fitness" and "Medical", following a careful examination of program type in different Android categories. To identify relevant web-based programs, a systematic search was conducted on September 5, 2016, using a Google Search query per condition (e.g., depression, smoking cessation) paired with terms such as "free online" and "self-help". For each condition, we looked into the organic results found within the first two pages, as studies have indicated that a negligible portion of users go beyond the second page [3,4]. If a source referring to a list of programs was found among these organic searches, we included those programs as well. The lists created through the searches for mobile applications and websites were then screened by title in order to remove duplicates and exclude irrelevant programs (e.g., magazine). Programs with unclear titles were examined (by a person who was not one of the program quality raters) using Google Play or website home page prior to exclusion.

Using a randomization website [5], after a completion of training that included rating of twelve programs, twenty one eHealth programs were randomly selected for each of the four conditions: two Delivery Mediums (mobile/website) X two Clinical Aims (health-related behavior/mental health) – reaching a total of 84 programs. For example, during this process 21 *mobile* applications which target *health-related behavior* were randomly selected (not including the 3 apps per condition that were used during raters' training).

Search Terms Used to identify eHealth Intervention Programs

Websites (Google search)

Health Related Behaviors:

1. (free online)
2. (Diet or weight loss) OR (fitness or physical activity) OR (smoking cessation) OR (alcohol consumption or alcohol abuse) OR (healthy behaviors or healthy habits))
3. (program) OR (training) OR (plan)
4. #1 and #2 and #3

Note: searches ran separately based on OR rules in #2 and #3

Mental Health:

1. (free online)
2. (Depression) OR (Anxiety) OR (Well Being) OR (Mental Health)
3. (self help program) OR (treatment) OR (CBT)
4. #1 and #2 and #3

Mobile (Google Play store)

Health Related Behaviors:

(Diet or weight loss) OR (fitness or physical activity) OR (smoking cessation) OR (alcohol consumption or alcohol abuse) OR (healthy behaviors or healthy habits)

Mental Health:

Depression OR Anxiety OR Mental-Health OR Well-being

Note: searches ran separately based on OR rules

1. Liberati A, Altman DG, Tetzlaff J, et al. The PRISMA statement for reporting systematic reviews and meta-analyses of studies that evaluate health care interventions: Explanation and elaboration. *Ann Intern Med*. 2009;151(4):W-65-W-94. PMID:19622512.
2. Mokdad AH, Marks JS, Stroup DF, Gerberding JL. Actual causes of death in the United States, 2000. *JAMA*. 2004;291(10):1238-1245. PMID:15010446.
3. Van Deursen AJ, Van Dijk JA. Using the Internet: Skill related problems in users' online behavior. *Interacting With Computers*. 2009;21(5):393-402.
4. iProspect. Search engine user behavior study: a white paper. 2006.
5. Random generator. https://www.random.org/lists/. Accessed December 22, 2016. Archived by WebCite® at http://www.webcitation.org/6mwaCaO2t
