# Supplementary material for: Examining Predictors of Real-World User Engagement with Self-Guided eHealth Interventions: Analysis of Mobile Apps and Websites Using a Novel Dataset
Source: J Med Internet Res. 2018 Dec 14;20(12):e11491. doi: 10.2196/11491 (PMC6315225; doi:10.2196/11491)
Supplement: Multimedia Appendix 2 [file jmir_v20i12e11491_app2.pdf]

Multimedia Appendix 2 – Description of Scales and Scale Items

Concepts	Description
**Quality Assessment Section**	
Usability	Assesses the ease of learning how to use the eHealth intervention Program (EHP) and the ease of utilizing it properly.
Visual Design	Assesses the look and feel of the program, the visual quality of the Graphical User Interface (GUI).
User Engagement	Assesses the extent to which the EHP's design attracts users to utilize it.
Content	Assesses the content provided or learned while using the EHP.
Therapeutic Persuasiveness	Assesses the extent to which the EHP is designed to encourage users to make positive behavior changes OR to maintain positive aspects of their life.
Therapeutic Alliance	Assesses the ability of the program to create an alliance with the user in order to effect a beneficial change.
**Checklists Section**	
Credibility Checklist	Assesses the degree to which the product / developers can be trusted.
Evidence Based Program	Assesses the quality of empirical research supporting the program's efficacy (a separate measure that is part of program's Credibility).

ENLIGHT – Quality Assessment Section

Usability

Assesses the ease of learning how to use the eHealth intervention Program (EHP) and the ease of utilizing it properly.

Note: Slow speed of operation and apparent errors should be reflected in all three items. Serious bugs/malfunctions mean Usability=1.0

A. Navigation. Is it easy/natural/frictionless to navigate through the EHP?

Note: Pay attention to how easy it is to (a) move from one location to another (if needed), and (b) move backwards.

1. **Very poor.** It is very difficult to move from one place to another. Many features are, therefore, not accessible when needed.
2. **Poor.** It is difficult to move from one place to another, making some features somewhat hard to reach when needed.
3. **Fair.** Navigation is okay, but not smooth.
4. **Good.** It is simple/natural to navigate through the EHP flow (but not ideal).
5. **Very good.** It is very clear how to navigate through the EHP and to access every desired / relevant location when needed.

B. Learnability. How easy is it to learn how to use the EHP at first? Is it self-explanatory?

Note: Consider complexity. Some programs are very complex and so might only score a maximum of 4.

1. **Very poor.** It takes too much time to learn how to use the program.
2. **Poor.** It takes a considerable amount of time to learn how to use the program. Only highly motivated users will take the time to learn it OR supplementary support is needed.
3. **Fair**. Users can learn how to use the program without additional support. Only a few of the important features require a considerable amount of time to learn.
4. **Good**. Learning to use the program is easy (but not ideal). Appropriate explanations appear if needed. None of the important features require a considerable amount of time to learn.
5. **Very good**. Learning to use the program is very easy, natural, and frictionless.

C. Ease of Use. How easy is it to use the EHP? Does the operator need to exert only the least possible effort to activate the desired features?

1. **Very poor.** The user has to exert a lot of effort that would have been unnecessary had the program been designed differently.
2. **Poor.** Utilizing key parts of the program demands effort from the end user.
3. **Fair.** Utilizing some parts of the program demands effort from the end user.
4. **Good.** Utilization could have been made more effortless by designing one (not major) feature differently.

5. **Very good.** The design best minimizes the effort required from the user.

Visual Design

Assesses the look and feel of the program, the visual quality of the Graphical User Interface (GUI).

A. **Aesthetics.** Is the visual design of the program attractive and appealing to its target audience? Does the program have a harmonious look and feel (including colors and fonts)?

1. **Not attractive at all.** The choice of colors/fonts/background is very poor.
2. **Not attractive.** The choice of colors/fonts/background does not make sense; however, some things are still adequately designed.
3. **Fair.** The program design makes some sense, but it is not attractive.
4. **Attractive.** Most parts of the program are attractive, but could be better.
5. **Very attractive.** Program design is well thought-out, and the program has a harmonious look and feel.

B. **Layout.** Does the EHP appear well-organized? Relate to: structure (e.g., pattern, consistency); how well it displays (only necessary) elements on screen; and whether important information is clear and stands out.

1. **Very poor.** The basic layout is completely wrong and disorganized. Frames don't fit the screen, and important parts are not featured.
2. **Poor.** The basic layout is poor and disorganized, but some aspects are adequate.
3. **Fair.** For the most part, relevant elements appear on the screen and more important aspects are featured. However, there are still some issues with the program's structure and organization.
4. **Good**. In addition to ensuring the relevance and salience of key aspects, the program is also structured and organized.
5. **Very good**. The GUI is very well organized and prioritized. Elements are displayed appropriately.

C. **Size.** Are the sizes of fonts/buttons/menus appropriate (for the target audience)? Can the size be changed if needed?

1. **Very poor**. All fonts/features are inappropriately sized. Things do not fit the screen (navigation / scrolling is needed) for no apparent reason.
2. **Poor**. The main fonts/features are inappropriately sized.
3. **Fair**. There is at least one key place where the size is wrong.
4. **Good**. Most of the fonts/buttons/menus are appropriately sized, but there is still a (non-salient) place where the size is wrong.
5. **Very good**. All fonts/buttons/menus are appropriately sized and well thought-out.

User Engagement

Assesses the extent to which the EHP's design attracts users to utilize it.

A. **Content Presentation**. Is the content presented in an engaging/interesting way (e.g., contains the right mix of video/audio/text/graphics)?

1. **Very poor.** The content is presented in the wrong way. For example, there is text throughout the program where narration would be more appropriate.
2. **Poor.** For the most part, the content is presented poorly, but some areas of presentation are adequate.
3. **Fair.** Some major areas are presented appropriately (e.g., via text or audio), but better ways to present the content are still needed.
4. **Good.** Content is delivered through the appropriate use of features, but something is still lacking.
5. **Very good.** The way the content is presented is well suited to the user's need and context (e.g., platform or time of use).

B. **Interactive.** Does the EHP include high-quality interactive features (which enable user input and reaction)?

1. **Very poor.** There are no interactive features.
2. **Poor.** There are few interactive features, or the interactive features are of poor quality.
3. **Fair.** There are interactive features, but they are of mediocre quality.
4. **Good.** The program presents a good interactive experience (but something is still missing).
5. **Very good.** The program presents a high-quality interactive experience.

N/A – The EHP is not (highly) interactive, and so this is not an appropriate way to examine such a program, for example, a trigger-based intervention.

C. **Not Irritating.** Does the program avoid irritation in the user's experience (e.g., by controlling notifications/alerts/sounds or avoiding irritating colors/fonts/sounds/expressions)?

Note: Consider pop-up advertisements.

1. **Very poor**. It is annoying and irritating to utilize the program.
2. **Poor**. Some of the program's key features are irritating.
3. **Fair**. There are some irritating features.
4. **Good**. For the most part, the EHP is not irritating, and users are able to modify any irritating aspects.
5. **Very good**. The program is not at all irritating, and, if relevant, users are given the opportunity to control potentially irritating aspects at the outset in order to avoid an irritating experience.

N/A – The EHP is not irritating, and this is not an appropriate way to examine such a program. For example, a program is very lean (e.g., absent of reminders that it should have)

and therefore does not have the potential to be irritating (i.e., giving it a high score would inappropriately skew the results).

D. **Targeted/Tailored/Personalized.** Are the features tailored to the usage context of the target group? If necessary, does the EHP enable the customization/personalization of the program (e.g., personalized goals/action items, online diary that maintains personal notes, choice of which content to receive)?

1. **Very poor**. The program does not have any targeted/tailored/personalized features.
2. **Poor.** The program includes a few targeted/tailored/personalized features.
3. **Fair**. The program incorporates a fair amount of targeting/tailoring/personalization.
4. **Good**. The program mostly provides a tailored/personalized experience based on users' needs.
5. **Very good**. The program is very well designed in terms of offering the user a targeted/tailored/personalized experience.

E. **Captivating.** Does utilizing the EHP pique the user's curiosity and interest (i.e., attract users to use it as needed)?

1. **Very poor.** The program is extremely boring and not desirable to use.
2. **Poor.** For the most part, the program features are boring, but there are some positives.
3. **Fair.** The program is neither boring, nor captivating.
4. **Good.** The program is interesting to use.
5. **Very good.** The program is highly attractive and piques the user's curiosity, excitement, and interest.

Content

Assesses the content provided or learned while using the EHP.

Note: As features (e.g., games) are a way of delivering information, the content conveyed within them should be examined.

A. **Evidence-Based Content**. Is the information provided accurate? Are there evidence-based techniques relevant for achieving the desired clinical aim of the program?

1. **Very poor**. The features/content do not reflect any evidence-based principles in this field.
2. **Poor**. The presentation of evidence-based techniques is sparse OR the program content is not very accurate.
3. **Fair**. There is some presentation of evidence-based techniques, and the content is mostly accurate.
4. **Good**. The program content is accurate and reflects evidence-based techniques (but is still not ideal).
5. **Very good.** The program content is accurate and based on sound evidence-based principles relevant to the clinical aim.

B. **Quality of Information Provision.** Is the information provided in a clear and appropriate way for the target audience?

Note: Users' age and cognitive and emotional abilities should be taken into account.

1. **Very poor.** None of the information is provided in a way that is clear or appropriate for the target audience.
2. **Poor.** Some of the information is provided in a clear and appropriate way.
3. **Fair.** The information is provided in a way that is mostly clear and appropriate, but could be better.
4. **Good.** The information is provided in a way that is clear and appropriate for the target audience, but still not ideal.
5. **Very good.** The information is provided in the most clear and appropriate way for the target audience.

C. **Complete and Concise**. Is there sufficient information throughout the program without any omissions, over-explanations, or irrelevant data?

1. **Very poor.** There is too much content that does not allow the user to grasp the relevant information, OR there is almost no content.
2. **Poor.** There is a great deal of content that interferes with the relevant information, OR the content is sparse.
3. **Fair.** There is some superfluous information, OR there are some omissions.
4. **Good.** The information is complete, but not concise enough, OR the information is concise, but not entirely complete.
5. **Very good.** The content is as complete and concise as it can be.

D. Clarity about the Program's Purpose. Is there sufficient and accurate information about the target audience, the clinical aim (e.g., potential outcomes), and appropriate ways to utilize the program (e.g., adjunct, standalone)?

Notes: Includes who should not use it; could be described in distribution channels such as app stores.

1. **Very poor.** There is no information at all about the program's purpose. / Information is either inappropriate or inaccurate.
2. **Poor.** There is little information or poor accuracy.
3. **Fair.** There are some explanations as to the program's purpose, but these might be insufficient for some users.
4. **Good.** The EHP explains who should use the program, what its purpose is, and how it should be utilized, but some information is still lacking.
5. **Very good.** The EHP provides a thorough explanation of who should use the program, what its purpose is, and how it should be utilized.

Therapeutic Persuasiveness

Assesses the extent to which the EHP is designed to encourage users to make positive behavior changes OR to maintain positive aspects of their life.
Note: Factors of social support (e.g., influence, facilitation, cooperation, recognition) should be taken into account while rating.

A. **Call to Action.** Does the EHP saliently set up measurable and relevant therapeutic activities and inspire/encourage users to complete them?

   Notes: Includes sending out prompts if appropriate; does the user have to take part in the goal-setting for the desired action(s) to be relevant/agreeable in this program? If so, rate accordingly.

   1. **Very Poor.** Action items are vague, implied, hidden, or non-existent.
   2. **Poor.** Some action items exist, but the EHP doesn't inspire users at all
   3. **Fair.** There are some relevant/targeted action items, and there is some degree of inspiration/encouragement.
   4. **Good.** For the most part, there are relevant/targeted action items and the program stimulates/inspires users to meet their goals.
   5. **Very good.** The desired therapeutic activities are well targeted, and the program clearly stimulates/inspires users to complete the activities.

B. **Load Reduction of Activities.** Are the therapeutic activities that users are required to complete sufficiently simple? Do the features of the EHP make it as easy as possible for users to complete the activities?

   Note: This is related also to the features that support the completion of activities.

   1. **Very poor**. The therapeutic activities are too complicated for target users to carry out AND the program features do not make the activities easier to complete.
   2. **Poor**. The activities are somewhat simplified, but the program features do not make the activities easier to complete.
   3. **Fair**. The activities are fairly simple and there are some features that make it easy for users to achieve their goals.
   4. **Good**. The activities are simple and straightforward, and the program features make it easy for users to achieve their goals (but still not ideal).
   5. **Very good**. The activities are as simple and relevant as possible, and the program features make it as easy as possible for users to engage in the therapeutic activities by providing them with the relevant tools “in house”.

C. **Therapeutic Rationale and Pathway.** Is the therapeutic pathway clear? Is it clear how working through each action item provided by the EHP should lead to the desired therapeutic outcome(s)?

Note: This should also be considered from the user's perspective.

1. **Very poor**. Users are asked to engage in activities without the therapeutic pathway being defined. The relationship between the activities and the desired outcome does not make sense.
2. **Poor**. While the relationship between the activities and therapeutic progress is understood, it is not clear how the EHP design and the way the action items are provided should lead to the desired therapeutic outcome.
3. **Fair**. It is somewhat clear how the EHP design and the way the action items are provided should lead to the desired therapeutic outcome.
4. **Good**. It is clear how the EHP design and the way the action items are provided should lead to the desired therapeutic outcome (but still not ideal).
5. **Very good**. It is very clear how the EHP design and the way the action items are provided should lead to the desired therapeutic outcome.

D. **Rewards.** Does the technology recognize desirable achievements and provide appropriate recognition?

Note: This includes documentation of "therapeutic investments," i.e., beneficial work done by the user that is documented in the program in a way that makes users want to stay committed to this pathway (e.g., acquiring points/badges for beneficial activities and showing them on a community board).

1. **Very poor.** The system does not reward users at all.
2. **Poor.** The system uses rewards sparsely/inappropriately.
3. **Fair.** The frequency/appropriateness of rewards is only fair.
4. **Good.** The technology pays attention to desirable achievements. There are rewards most of the time, but they are not ideal (e.g., the same rewards are used all the time, too many rewards, or rewards not creative/accurate enough).
5. **Very good.** The system does a very good job acknowledging when users reach desirable achievements and rewarding them appropriately/creatively/accurately.

E. **Real Data-Driven / Adaptive Content.** Is the program content influenced by the real user's state and/or achievements? *Examples: Content becomes available when the user is ready (i.e., has made appropriate progress); program content changes based on the user's real behavior/success/failures.*

Note: The user's state does not have to rely on self-assessment; other methods could include passive sensing and clinicians' input.

1. **Very poor**. The user's progress is not monitored, and content is available regardless of the user's state.
2. **Poor**. The user's progress is not well monitored, and content mostly disregards the user's state.
3. **Fair**. The user's progress is monitored but not in a way that has a strong impact on program content, OR the program is adaptive, but not based on an accurate evaluation of the user's state.

4. **Good**. The EHP appropriately monitors the user's state and relies somewhat on the user's progress to determine content.
5. **Very good**. The program adapts well to the user's state/progress by changing its available content accordingly.

F. Ongoing Feedback. Does the program provide appropriate <u>ongoing</u> feedback on the user's state?

1. **Very poor.** The program does not provide any feedback.
2. **Poor.** The program provides minimal feedback, for example, only after enrollment and taking baseline measurements.
3. **Fair.** Feedback is embedded within the program (e.g., graphs of outcome measures, calorie intake), but not in a way that provides users with a good understanding of their state.
4. **Good.** Feedback is embedded within the program, mainly in a way that provides users with an understanding of their state (e.g., via clear verbal explanation).
5. **Very good.** Feedback is embedded within the program with salient, accurate, and appropriate regard to the user's current state.

G. Expectations and Relevance. Does the program <u>convincingly advocate</u> for intervention's relevance, and explain the intervention framework and the general expectations of the user?

Note: Advocating entails relating to one's own state, difficulties in making/sustaining a change, motivation and consequences for using it.

1. **Very poor.** There is no explanation of the program's relevance and its expectations of the user.
2. **Poor.** The program offers only limited explanation of its relevance and expectations of the user.
3. **Fair.** The program offers an adequate explanation of its relevance and expectations of the user.
4. **Good.** The EHP advocates for its relevance, and explains the framework and general expectations appropriately (but something is still missing).
5. **Very good.** The EHP effectively advocates for its relevance, and explains the framework and general expectations.

N/A – The EHP does not explain its expectations/relevance, but this is not an appropriate way to examine such a program. For example, the targeting of a program makes it irrelevant to set up expectations.

Therapeutic Alliance

Assesses the ability of the program to create an alliance with the user in order to effect a beneficial change.
Note: Factors of social support (e.g., influence, facilitation, cooperation, recognition) should be taken into account while rating.

A. Basic Acceptance and Support. Does the EHP make an effort to show that it understands and empathizes with the user; genuinely cares for the user; and relates to the user in a positive fashion?

Note: The EHP is not a person so this should be done appropriately within the limits of the medium.

1. **Very poor (neutral).** There is no positive regard for OR effort to understand the user's perspective.
2. **Poor.** There is only a minimal gesture to demonstrate understanding/caring for the user's perspective.
3. **Fair.** In general, there is positive regard and care for the user (some degree of outreach is needed to receive 3).
4. **Good**. The EHP is designed to provide users with feelings of basic acceptance and support.
5. **Very good.** The EHP proactively shows users that they are accepted and supported as a salient aspect of the program.

B. Positive Therapeutic Expectations. Does the EHP encourage users to expect beneficial outcomes from utilizing the program and to rely upon it in the medical context?

Note: Consider how well the program instills confidence in users that they are in "good hands" (projecting trustworthiness and professionalism through tone, narrative, convincing presentation, reliable "look and feel", and meeting people's exact needs at the right time).

1. **Very poor (neutral).** The program does not instill confidence in users that they will benefit from the program. No professionalism/trustworthiness is conveyed.
2. **Poor.** The program instills minimal confidence in the user and conveys limited professionalism/trustworthiness.
3. **Fair.** The program instills some confidence in the user and conveys some professionalism/trustworthiness.
4. **Good.** The program instills a good degree of confidence in the user and conveys a good degree of professionalism/trustworthiness, but something is still missing.
5. **Very good.** The program effectively instills confidence in users that they will benefit from the program through professionalism and trustworthiness.

C. Relatability. Does the EHP offer a good representation of a human factor that is easily relatable within the therapeutic context/process? Examples include a professional character who directs the user throughout the program; a peer who was in a similar situation and is now better (e.g., fitness); a vivid virtual character who leads the user; a community of people working together for change.

Notes: A community of people NOT "working" to positively support each other does not count; even text messages could create such projections through language, sender's identity, and responsiveness.

1. **Very poor.** There is no relatable human factor.
2. **Poor.** Some representation of a human factor exists, but it is not really therapeutic or easily relatable.
3. **Fair.** There is a representation of a positive human factor, but no effort is made to communicate with the user on a personal level. The human factor seems somewhat distant from the user.
4. **Good.** There is a representation of a human factor that users can relate to throughout the therapeutic process. However, users might not be able to relate to this factor in an ideal way.
5. **Very good.** The representation of a human factor is salient throughout the therapeutic process; for example, users are potentially able to become really familiar with this human factor (e.g., professional character) or feel they are part of a community.

Note: *When presenting/calculating only Therapeutic Alliance concept (without other concepts), the following items should be included: Therapeutic Rationale and Pathway & Targeting/Tailoring/Personalization.*

ENLIGHT - CHECKLISTS SECTION

The checklists below are based on acknowledged criteria that cover distinct domains related to program use. These checklists do not directly impact the end user's experience of the ehealth intervention program's efficacy, but may expose the user (or provider) to acknowledged risks.

Credibility Checklist

Assesses the degree to which the product / developers can be trusted. The information related to the Credibility criteria should be easily accessible (e.g., on the program's website or mobile app) and not require a comprehensive search (not known=no).

Suggested interpretation of the checklist:

Accumulate points and add +1

1 = Can't be accounted for; 2 = Poor; 3-4 = Fair; 5 = Good; 6-7 = Very good; 8 = Excellent

1. **Owners' Credibility;** Does the app come from a legitimate source?

   - ☐ **Yes (=1), if both conditions are satisfied:**
     - Registered company/institute with more than 5 years of specific experience in the eHealth field OR academic institution (e.g., university) OR health care system (or large health providers' organization). *Note: Can't be an individual (must be a registered entity).*
     - Contact details of the company are available, easy to find, and include office address, email, and team.
   - ☐ **No (=0).** One of the above is not true.

2. **Maintenance***

Frequency of update, Maintenance of site.

Last update – less than 6 months.

- ☐ Yes (=1)
- ☐ No (=0)

* Without data from a third party this item cannot be examined for websites
(in that case add 1 point for websites)

3. **Strong Advisory Support**

Strong advisory support with clinical/design/development team able to lead the product design (not known=no; cannot be just a licensed clinician; must be someone with established experience in designing such products for the relevant clinical aim).

Notes: Beware of self-appointed experts. Identification process: preferably on site or on a mother website, but has to be people who take ownership of the specific program; it is possible to identify the developers/owners in research literature.

- ☐ 2= A leading expert is part of the team OR more than one leading expert are part of its advisory

board (e.g., associate professors who specifically develop these kinds of products in their career)

- ☐ 1= Clinical and design experts are part of the team/advisory board (e.g., junior researchers in the field, certified clinicians with experience in the field)
- ☐ 0= No

4. Third-Party Endorsement

Product has been verified, given a good review, and endorsed by a legitimate/reliable source (e.g., APA; FDA; SAMSHA; NIH; NHS in the UK; NICE in the UK).

Notes: Blog/paper publishing is not an endorsement; funding of research is not an endorsement; the source has to recommend that its members/affiliates use the product or ask their physicians to provide referrals to this program. Legitimate/Reliable Source: Must be a source with acknowledged core expertise in health (in most cases NASA would not be considered a reliable source).

- ☐ Yes (=2)
- ☐ No (=0)

5. Evidence of Successful Implementation

- ☐ Yes (=1) if one of the following is true:
    - Mobile: more than 100,000 downloads. Website: monthly average > 50,000 unique users (calculated over the last 4–12 months; desired examination period is based on available data but has to be consistent across programs).
    - Mobile: Between 20,000-100,000 downloads; Website > 10,000 unique monthly users. AND high user engagement with low retention rates examined by a third party (desired period of use and number of users can be changed based on the program's clinical aim).
    - Implemented within: health system under usual care OR large group of clinicians (>1,000) officially refers patients to utilize it.
    - Over 1,000 reviews > 4.0
- ☐ No (=0).

Evidence-Based Program

Assesses the quality of empirical research supporting program's efficacy.

1. **Very poor.** There is no research on the program OR poor (#2) research was done only by the developers (who are NOT active researchers working within a not-for-profit institute).
2. **Poor.** The research conducted is of low quality, for example, relying on users' attitudes toward using the program, or not conducted within this technology zeitgeist.
3. **Fair.** The research is of fair quality and was conducted within this technology zeitgeist. For example, it involved at least two pilots with the right outcome measurement OR one RCT, but did not utilize the most reliable/valid outcome measurement (e.g., not a pre-registered clinical trial, not a validated outcome tool).
4. **Good.** Some good research shows evidence of efficacy. For example, there have been several published pilot studies + one pre-registered RCT with sufficient power done by a

credible source showing it is superior to wait list control condition OR one pre-registered RCT at the level of RCTs described in 5. Related research was conducted within this technology zeitgeist.

5. **Very good.** The program boasts strong research support, with at least two pre-registered RCTs with adequate statistical power conducted by at least two different credible sources, in which: the program was found to be superior to an appropriate placebo (wait list is not a placebo) or equivalent to acceptable evidence-based treatment groups. Related research was conducted within this technology zeitgeist.

Note: RCT – Randomized Controlled Trial
