# Supplementary material for: Examining Predictors of Real-World User Engagement with Self-Guided eHealth Interventions: Analysis of Mobile Apps and Websites Using a Novel Dataset
Source: J Med Internet Res. 2018 Dec 14;20(12):e11491. doi: 10.2196/11491 (PMC6315225; doi:10.2196/11491)
Supplement: Multimedia Appendix 3 [file jmir_v20i12e11491_app3.pdf]

Multimedia Appendix 3 - Included Programs

Program's Name	Website	Mobile App	Website URL	Google Play Store (Link)
5 Days Weight Loss Plan!		1		https://play.google.com/store/apps/details?id=com.andromo.dev429255.app398273
7 Cups Of Tea	1	1	https://www.7cups.com	https://play.google.com/store/apps/details?id=com.sevencupsoftea.app
Be Mindful online	1		https://www.bemindfulonline.com	
Beating Bipolar	1		http://beatingbipolar.org/	
Bliss		1	http://bliss31.com/	https://play.google.com/store/apps/details?id=com.bliss.phonegap&hl=en
Booster Buddy		1		https://play.google.com/store/apps/details?id=com.viha.boosterbuddy&hl=en
BrainHQ	1	1	http://www.brainhq.com/welcome	https://play.google.com/store/apps/details?id=com.positscience.brainhq.app
Breath Better	1		https://www.breathebetter.me	
Breathe2Relax		1		https://play.google.com/store/apps/details?id=org.t2health.breathe2relax&hl=en
Calm	1	1	https://www.calm.com/	https://play.google.com/store/apps/details?id=com.calm.android&hl=en
Calm Harm		1	http://www.stem4.org.uk/	https://play.google.com/store/apps/details?id=uk.org.stem4.calmharm&rdid=uk.org.stem4.calmharm
Daily Challenge	1	1	https://challenge.meyouhealth.com/signup/	https://play.google.com/store/apps/details?id=com.meyouhealth.dailychallenge&referrer=utm_source%3Ddailychallenge%26utm_medium%3Demail

Program’s Name	Website	Mobile App	Website URL	Google Play Store (Link)
Daybreak - Drink less		1	https://www.hellosundaymorning.org/daybreak/#about-app	https://play.google.com/store/apps/details?id=com.hellosundaymorning.android.challenges&hl=en?utm_source=daybreak2
Depression CBT Self-Help Guide		1		https://play.google.com/store/apps/details?id=com.excelatlife.depression
Depression Center	1		http://www.depressioncenter.net/Default.aspx	
Diet plan weight loss		1		https://play.google.com/store/apps/details?id=diet.dietasparaadelgazar
Down Your Drink	1		https://www.downyourdrink.org.uk/session/new	
Eat This Much	1		www.eatthismuch.com	
E-Couch	1		https://ecouch.anu.edu.au/welcome	
Fit Brains	1	1	http://www.fitbrains.com/	https://play.google.com/store/apps/details?id=com.vivitylabs.android.braintrainer&hl=en?utm_source=fitbrains_website
Free Trainers	1		https://www.freetrainers.com/account/login/	
HAPPYneuron	1		http://www.happy-neuron.com	https://play.google.com/store/apps/details?id=air.happyneuron.braintraining
Headspace		1	https://www.headspace.com/	https://play.google.com/store/apps/details?id=com.getsomeheadspace.android&hl=en
Health Lab		1		https://play.google.com/store/apps/details?id=com.tictrac.android.oned
Healthy Life Project	1		http://www.healthylifeproject.com.au/program/web/dashboard	

Program’s Name	Website	Mobile App	Website URL	Google Play Store (Link)
Aware-Meditation		1	https://my.awaremeditationapp.com/profile	https://play.google.com/store/apps/details?id=com.mindfulness.aware&referrer=utm_source%3Dwebsite%26utm_medium%3Dbutton&rdid=com.mindfulness.aware
InsightTimer		1	https://insighttimer.com/	https://play.google.com/store/apps/details?id=com.spotlightsix.zentimerlite2&rdid=com.spotlightsix.zentimerlite2
Joyable	1		https://joyable.com/	
Just Move - Fitness Motivation		1		https://play.google.com/store/apps/details?id=com.justmoveapp
Lantern		1	https://golantern.com/intake/#/get_app?_k=4cd6wa	https://play.google.com/store/apps/details?id=com.golantern.lantern.beta&rdid=com.golantern.lantern.beta
LG health		1		https://play.google.com/store/apps/details?id=com.lge.lifetracker
Life Reboot- Fight Depression		1		https://play.google.com/store/apps/details?id=com.photon.lifereboot
LightExistence		1		https://play.google.com/store/apps/details?id=com.dynamesproductions.montymason.lightexistence_depression_self_help&rdid=com.dynamesproductions.montymason.lightexistence_depression_self_help
Living Life to The Fullest	1		http://www.llttf.com/	
Lose weight without dieting		1		https://play.google.com/store/apps/details?id=ru.harmonicsoft.caloriecounter
Meditate OM		1		https://play.google.com/store/apps/details?id=com.panagola.app.om
MoodGYM	1		https://moodgym.com.au/	

Program’s Name	Website	Mobile App	Website URL	Google Play Store (Link)
moodmission		1	http://moodmission.com/	https://play.google.com/store/apps/details?id=com.moodmission.moodmissionapp&rdid=com.moodmission.moodmissionapp&pli=1
MoodTools - Depression Aid		1	http://www.moodtools.org/	https://play.google.com/store/apps/details?id=com.moodtools.moodtools
My Compass	1		https://www.mycompass.org.au/	
My Drink Control	1		http://www.mydrinkcontrol.ch/en/	https://play.google.com/store/apps/details?id=ch.mydrinkcontrol.app&hl=en
My Healthy Balance	1		https://myhealthybalance.com.au/Account/Login	
My Trainer Dasi		1		https://play.google.com/store/apps/details?id=com.donets.dietplan
myQuit Time		1		https://play.google.com/store/apps/details?id=com.tusnuadesigns.myquittime
Omvana	1	1	http://play.omvana.com/welcome	https://play.google.com/store/apps/details?id=com.omvana.mixer&hl=en
Pacifica		1	https://www.thinkpacifica.com/	https://play.google.com/store/apps/details?id=com.pacificalabs.pacifica
PE Coach		1		https://play.google.com/store/apps/details?id=org.t2health.pe
Peak		1		https://play.google.com/store/apps/details?id=com.brainbow.peak.app&referrer=adjust_reftag%3DcIbGLY66sV6Wx&rdid=com.brainbow.peak.app
PTSD Coach		1		https://play.google.com/store/apps/details?id=is.vertical.ptsdcoach

Program’s Name	Website	Mobile App	Website URL	Google Play Store (Link)
Pzizz - Deep Sleep & Power Nap		1	http://pzizz.com/	https://play.google.com/store/apps/details?id=com.pzizz.android&hl=enhttp://pzizz.com/
Qi Gong Meditation Relaxation		1		https://play.google.com/store/apps/details?id=com.excelatlife.motivation&hl=en
Quit Coach	1		http://www.quitcoach.org.au/	
Quit Smoking		1		https://play.google.com/store/apps/details?id=com.umtgrn.quitsmoking&hl=en
recovery record Eating Disorder Management	1	1	https://www.recoveryrecord.com/	https://play.google.com/store/apps/details?id=com.recoveryrecord
Rehapp		1		https://play.google.com/store/apps/details?id=com.rehapp
Rise Up + Recover		1	https://www.recoverywarriors.com/app/	https://play.google.com/store/apps/details?id=com.rur&hl=en
Self-Help for Anxiety Management		1	http://sam-app.org.uk/	https://play.google.com/store/apps/details?id=com.uwe.myoxygen&hl=en
Serene	1		http://serene.me.uk/kiosk-0/anxiety_menu.php	
Serenita		1	http://www.eco-fusion.com/serenita/	https://play.google.com/store/apps/details?id=com.ecofusion.mentally
Seven - 7 Minute Workout		1		https://play.google.com/store/apps/details?id=se.perigee.android.seven
Smiling Mind		1	https://smilingmind.com.au/our-programs/for-adults/	https://play.google.com/store/apps/details?id=com.smilingmind.app&hl=en
Start 2	1		http://www.start2.co.uk/	
Start Your Diet	1		http://www.startyourdiet.com/login.php	

Program’s Name	Website	Mobile App	Website URL	Google Play Store (Link)
Stop Breathe Think	1	1	https://app.stopbreathethink.org/	https://play.google.com/store/apps/details?id=org.stopbreathethink.app&referrer=af_tranid%3D6fNg7HSpZ6QfooaPh08nFA%26pid%3DWebsite%26c%3DHome_Top_Button
Stop Smoking In Two Hours		1		https://play.google.com/store/apps/details?id=com.juicemaster.stopSmokingIn2Hours
StopPulling	1		https://stoppulling.com	
SuperBetter	1	1	https://www.superbetter.com/	https://play.google.com/store/apps/details?id=com.superbetter.paid
The Desk	1		https://www.thedesk.org.au	
Vet Change	1		https://vetchange.org/home/index2	
Weight Loss for Beginners		1		https://play.google.com/store/apps/details?id=com.weight.loss.beginners1234
WellMind		1		https://play.google.com/store/apps/details?id=com.bluestepsolutions.wellmind
Wildflowers Mindfulness		1	http://www.wildflowersmindfulness.com/	https://play.google.com/store/apps/details?id=com.mobiointeractive.wildflowers
Woebot		1	https://woebot.io/	https://play.google.com/store/apps/details?id=com.woebot
Worry Box		1		https://play.google.com/store/apps/details?id=com.excelatlife.worrybox
Wysa		1		https://play.google.com/store/apps/details?id=bot.touchkin&rdid=bot.touchkin
